# Calming the Storm: Natural Immunosuppressants as Adjuvants to Target the Cytokine Storm in COVID-19

**DOI:** 10.3389/fphar.2020.583777

**Published:** 2021-01-27

**Authors:** Angela E. Peter, B. V. Sandeep, B. Ganga Rao, V. Lakshmi Kalpana

**Affiliations:** ^1^ Department of Biotechnology, College of Science and Technology, Andhra University, Visakhapatnam, India; ^2^ Andhra University College of Pharmaceutical Sciences, Andhra University, Visakhapatnam, India; ^3^ Department of Human Genetics, College of Science and Technology, Andhra University, Visakhapatnam, India

**Keywords:** COVID-19, cytokine storm, immunomodulatory agents, plant-derived immunosuppressants, adjuvant

## Abstract

The COVID-19 pandemic has caused a global health crisis, with no specific antiviral to treat the infection and the absence of a suitable vaccine to prevent it. While some individuals contracting the SARS-CoV-2 infection exhibit a well coordinated immune response and recover, others display a dysfunctional immune response leading to serious complications including ARDS, sepsis, MOF; associated with morbidity and mortality. Studies revealed that in patients with a dysfunctional immune response, there is a massive cytokine and chemokine release, referred to as the ‘cytokine storm’. As a result, such patients exhibit higher levels of pro-inflammatory/modulatory cytokines and chemokines like TNFα, INFγ, IL-1β, IL-2, IL-4, IL-6, IL-7, IL-9, IL-10, IL-12, IL-13, IL-17, G-CSF, GM-CSF, MCSF, HGF and chemokines CXCL8, MCP1, IP10, MIP1α and MIP1β. Targeting this cytokine storm is a novel, promising treatment strategy to alleviate this excess influx of cytokines observed at the site of infection and their subsequent disastrous consequences. Natural immunosuppressant compounds, derived from plant sources like curcumin, luteolin, piperine, resveratrol are known to inhibit the production and release of pro-inflammatory cytokines and chemokines. This inhibitory effect is mediated by altering signal pathways like NF-κB, JAK/STAT, MAPK/ERK that are involved in the production and release of cytokines and chemokines. The use of these natural immunosuppressants as adjuvants to ameliorate the cytokine storm; in combination with antiviral agents and other treatment drugs currently in use presents a novel, synergistic approach for the treatment and effective cure of COVID-19. This review briefly describes the immunopathogenesis of the cytokine storm observed in SARS-CoV-2 infection and details some natural immunosuppressants that can be used as adjuvants in treating COVID-19 disease.

## Introduction

The coronavirus disease 2019 (COVID-19) caused by a novel *ß*-coronavirus, SARS-CoV-2 was first reported in Wuhan, China in December 2019 (Wang et al., 2020a). Spreading rapidly across the globe, the outbreak was declared as a Public Health Emergency of International Concern on 30 January 2020 by the World Health Organization (World Health Organization, 2020a). The reality and challenge of the present situation is that there is no specific drug to treat and cure the disease; neither is there a vaccine for prevention from it (at the time of submission of this review) (World Health Organization, 2020b).

One characteristic feature of the COVID-19 disease is the complex immune dysregulation observed in patients. This immune dysfunction causes deleterious clinical manifestations that lead up to organ injury, consequent organ failure and ultimately mortality (Giamarellos-Bourboulis et al., 2020; Li et al., 2020a). The hallmark of the immune dysregulation observed is an exaggerated immune response; manifested as hyperinflammation and hypercytokinemia (cytokine storm syndrome/ cytokine storm) in the COVID-19 patients. The factors that contribute to the state of hyperinflammation are persistent lymphopenia, neutrophilia, over-activation of complement components C3, C3a, C5, C5a and mannose binding lectin-associated serine protease (MASP2); in addition to the cytokine storm (Girija et al., 2020). The cytokine storm observed is the most dangerous and potentially life-threatening event in the COVID-19 disease. This is because it plays a crucial role in disease aggravation by promoting acute respiratory distress syndrome (ARDS) and multiple organ failure (MOF) (Coperchini et al., 2020; Ye et al., 2020). Several studies suggest that in addition to the use of antiviral drugs for the treatment of COVID-19, downregulation of the cytokine storm would prove to be an efficient treatment strategy to successfully combat the disease (Rahmati and Moosavi, 2020; Soy et al., 2020; Zhao, 2020). Plants, plant extracts and their derivatives have been known to possess immunomodulatory properties. Additionally, some plant-derived bioactive compounds have been evaluated for their ability to suppress the cytokine storm associated with inflammation and disease. This paper discusses the cytokine storm syndrome observed in COVID-19 patients in brief and emphasizes on some natural immunosuppressant agents derived from plants sources that can play an important role in targeting and mitigation of the cytokine storm observed in COVID-19.

## COVID-19 and the Cytokine Storm

### Introduction to COVID-19

COVID-19 is a viral disease caused by a beta coronavirus, SARS-CoV-2; an enveloped, non-segmented RNA virus (Astuti and Ysrafil, 2020; Wang et al., 2020b). The disease outbreak, declared as an ongoing pandemic by the WHO is widespread; affecting 220 countries, areas/ territories across the world with a total of 55,928,327 confirmed cases and with 1,344,003 confirmed deaths reported (World Health Organization, 2020c). Transmission of SARS-CoV-2 occurs primarily through respiratory droplets and contact routes (World Health Organization, 2020d). The spike protein plays an important role for SARS-CoV-2 to gain access to the intracellular compartment of the host. The receptor-binding domain (RBD) of the S1 subunit of the spike protein binds to the hACE2 receptor with high affinity, facilitating viral entry and infection in the lower respiratory tract cells (Hemmat et al., 2020; Shang et al., 2020). Once SARS-CoV-2 enters the host cell, its RNA is translated and viral replication for the production of mature newly synthesized virons occurs, which are released out of the infected cells (Shereen et al., 2020). The mean incubation period for SARS-CoV-2 is 3–7 days (Li et al., 2020b). Symptoms develop post the incubation period (Wujtewicz et al., 2020). Some infected individuals are asymptomatic, others are symptomatic with mild disease and, a third group is symptomatic with severe disease (Tabata et al., 2020). The symptoms of the disease are fever, cough, fatigue, headache, myalgia, sore throat, shortness of breath, sputum production and diarrhea. Other symptoms include chest pain, chills, nasal congestion, rhinorrhea and nausea (Fu et al., 2020a). Clinical presentations include elevated levels of lactate dehydrogenase, creatine kinase, alanine transaminase and aspartate aminotransferase (Wang et al., 2020a). Lymphocytopenia, high exhaustion levels and reduced functional diversity of T-cells (CD4^+^ and CD8^+^ T-cells) in peripheral blood is also another clinical feature of COVID-19 patients (Zheng et al., 2020a; Zheng et al., 2020b; Zhao et al., 2020). Higher levels of serum IgM, IgG and IgA can be detected in patients; severely ill SARS-CoV-2 patients have significantly higher antibody levels than mildly ill patients (Ma et al., 2020). The platelet and neutrophil levels are also elevated in patients (Zhou et al., 2020). Elevated D-dimer levels, prolonged prothrombin time (PT) and altered levels of fibrinogen observed point to the state of pulmonary intravascular coagulopathy that develops in patients (Zhang et al., 2020a; Lancet Haematol, 2020; Connors and Lavy, 2020; Long et al., 2020; McGonagle et al., 2020). Severe COVID-19 patients exhibit widespread complement activation which is characterized by C3 activation, C3a generation, C3 fragment deposition and increased serum C5a levels (Mastaglio et al., 2020; Noris et al., 2020; Risitano et al., 2020). Inflammatory markers like erythrocyte sedimentation rate (ESR), IL-6, C-reactive protein (CRP) and procalcitonin (PCT) are also elevated; especially in the patients with severe disease (Zeng et al., 2020). Elevated levels of all these inflammatory markers have been associated with disease severity; indicating the hyper-immune inflammatory state existing in the body resulting in higher morbidity and mortality in this patient group (Chen et al., 2020a; Chen et al., 2020b; Lippi and Plabani, 2020; Sun et al., 2020). Patients also exhibited higher levels of pro-inflammatory cytokines TNFα, IFNγ, IL-1β, IL-2, IL-4, IL-6, IL-7, IL-9, IL-12, IL-13, IL-17, G-CSF, GM-CSF, MCSF and chemokines CXCL8, MCP1, IP10, MIP1α, MIP1β. As in the case of inflammatory markers, the levels of serum cytokines and chemokines are higher in patients with severe disease. A diverse cytokine profile is observed in the two groups of patients; with critically ill patients showing higher levels of IL-1, IL-6, IL-2, IL-7, IL-10, IL-17, G-CSF, IP10, MCP1, MIP1α and TNFα. Among the cytokines, IL-6, IL-10 and CXCL10 are predictive of high risk patients of disease deterioration. As a consequence of the surging cytokine levels, the epithelial lining of the lungs gets injured that leads to deterioration of the alveolar cellular barriers. Changes to the microvasculature are also observed owing to damage in the endothelial cells. These changes results in the development of ARDS (Schett et al., 2020a; Han et al., 2020; Laing et al., 2020; Lingeswaran et al., 2020; Lingeswaran et al., 2020). The immunopathology gradually progresses to respiratory failure, establishment of secondary infections, sepsis and septic shock, multiple organ failure (kidney, liver); an overall poor prognosis that can end up in mortality (Gabriella et al., 2020; Guo et al., 2020; Hou et al., 2020).

### Cytokine Storm/ Hypercytokinemia/ Cytokine Storm Syndrome

Cytokine storm and hypercytokinemia are the two terminologies used to describe a severe, life-threatening condition that can occur as a result of an infection, autoimmune condition, or other disease. The National Cancer Institute defines cytokine storm as a severe immune reaction in which the body releases too many cytokines into the blood too quickly (National Cancer Institute, 2020). This condition is also referred to as cytokine storm syndrome sometimes (Shimabukuro-Vornhagen et al., 2018). These cytokines are a diverse group of small, non-structural, low molecular weight protein signaling molecules that are secreted and released by cells that have a complex regulatory influence on immunity and inflammation (Zhang and An, 2007; Ray et al., 2016). They play a significant role in regulating the immune response in health and disease (Duque and Descoteaux, 2014). Escalated cytokine production is associated with disease conditions (Orzechowski et al., 2014). Although the clinical presentations of cytokines vary for different diseases, they are characterized by broadly similar cytokine profiles. Cytokines generally associated with cytokine storm are interferons, interleukins, chemokines, colony stimulating factors (CSFs) and tumor necrosis factors (TNFs) (Tisoncik et al., 2012).

### Immunopathogenesis of Cytokine Storm in COVID-19

The immune system plays a crucial role in the control and resolution of SARS-CoV-2 infection. An out-of-control immune response to the pathogen can lead to immunopathogenesis that can prove to be fatal; causing excessive inflammation and even death (Guo et al., 2020; Qin et al., 2020). In most of the individuals infected with SARS-CoV-2, the immune system is primed and cells are recruited which clears the infection in the lung. The immune response then gradually recedes and the patients recover successfully. However, there are some patients wherein a dysfunctional immune response is observed, that triggers a massive cytokine and chemokine release; ‘cytokine storm’, mediating widespread inflammation in the lung (Catanzaro et al., 2020; Tay et al., 2020). One of the major causes of the ARDS and MOF observed in severe SARS-CoV-2 infection is the cytokine storm (Lingeswaran et al., 2020; Ye et al., 2020). Studies revealed that the progression of SARS-CoV-2 infection is associated with cytokine storm; higher level of cytokine storm is associated with more severe disease development (Tang et al., 2020a; Han et al., 2020). Plasma levels of cytokines IL-1β, IL-1RA, IL-7, IL-9, IL-10, FGF, G-CSF, GM-CSF, PDGF, VEGF, IFNγ, TNF and chemokines CXCL8, IP10, MCP1, MIP1α, MIP1β are significantly increased in patients with COVID-19 compared to healthy individuals. Furthermore, pro-inflammatory cytokines IL-2, IL-7, IL-10, G-CSF, TNF and chemokines IP10, MCP1, MIP1α are increased in severe patients compared with mildly-infected patients (Tang et al., 2020b).

Cytokines and chemokines are essential immune system mediators that have a significant role to play in maintenance of anti-viral immunity (Melchjorsen et al., 2003; Sládková and Kostolansky 2006). SARS-CoV-2 activates both the innate and adaptive immune response (Catanzaro et al., 2020). Initially after the entry of the virus into host cells, pathogen recognition receptors (PRRs) like TLR7, TLR8, RLRs and NLRs expressed by epithelial cells and antigen presenting cells like alveolar macrophages facilitate the recognition of virus by recognizing PAMP compromised nucleic acids, carbohydrate moieties, glycoproteins, lipoproteins, intermediate products such as dsRNA and small molecules found in the structural components of virus. TLR3, TLR7 and TLR8 are the first to identify the virus and this is responsible for an enhanced interferon production. Viral recognition triggers the stimulation of the innate immune response that leads to the activation of several signal transduction cascades and consequent downstream transcription factors resulting in the expression of genes encoding pro-inflammatory cytokines and chemokines; the SARS-CoV-2 signature characterized by induction of IL-1Rα, IL-1β, IL-6 and TNF. The chemokines produced serves to attract more innate immune response cells; NK cells, dendritic cells, polymorphonuclear leukocytes and monocytes; which in turn produce chemokines; MIG, IP10 and MCP-1 that are capable of recruiting lymphocytes. These recruited T-lymphocytes serve to recognize the antigen that is presented by the dendritic cells. The primed CD4 T-cells then execute the highly specific adaptive immune response by giving the signal to the B-cells for the production of antibodies and to the cytotoxic CD8 T-cells that are capable of targeting and eliminating the SARS-CoV-2. The antibodies that are produced; mainly IgG, IgM and IgA are highly specific and are directed toward the SARS-CoV-2 surface glycoproteins; primarily the spike glycoprotein and the nucleocapsid protein. This constitutes the humoral immune response to SARS-CoV-2 and these antibodies are vital to neutralize the viral infection of the human cells and tissues expressing ACE2. The cellular immune response to SARS-CoV-2 is mediated by the T-helper cells; the CD4 T-cells that help B-cells produce neutralizing antibodies, the cytotoxic CD8 T-cells; aided by helper T-cells that directly kill SARS-CoV-2-infected cells, the other T-cells; including the Th17-cells that drive the subsequent inflammatory response observed and the regulatory T-cells that are responsible for containing the immune response to preventing an exaggerated response (García, 2020; Herb et al., 2020; Hotez et al., 2020; Poland et al., 2020; Ragab et al., 2020; Shah et al., 2020; Song et al., 2020; Soy et al., 2020; Tay et al., 2020; Vabret et al., 2020).

Studies have revealed that the transition from the innate to adaptive immune response is critical for the clinical progress of the SARS-CoV-2 infection. Though the immune regulatory events in play during this transition are poorly understood at this point, it is clear that it either leads to a protective immune response in some patients or a dysfunctional immune response in others. Another contributing factor is the dysregulated interferon responses. SARS-CoV-2 replication during the incubation period in the host cells is stealthy; without detectably triggering interferon production resulting in high viral load. As a result, there is an initial, suppressed interferon response in early stage of infection. In severe COVID-19 patients, however, robust IFN-1 responses have been reported. Such a dysfunctional immune response results in failure to effectively clear the pathogen and persistent viral replication ensuing an exacerbated inflammatory response and elevated cytokine production. Together, impaired viral clearance, low levels of type I interferons in the initial stage of infection, increased neutrophil extracellular traps and triggered NK cell activation constitute the plausible predisposing factors that drive the cytokine storm or cytokine storm syndrome in COVID-19 contributing to the aggressive inflammatory response. Other contributing factors to the cytokine storm syndrome include pyroptosis; an inflammatory form of programmed cell death and ACE2 receptor mediated-increase in cytokines IL-6 and MCP1 triggered by an increased angiotensin II following the disruption of the rennin-angiotensin system; caused when S1 subunit of spike protein binds to hACE2 in the alveolar macrophages (Acharya et al., 2020; Fu et al., 2020b; Golonka et al., 2020; Iwasaki et al., 2020; Lee and Shin, 2020; Lee et al., 2020; Mahmudpour et al., 2020).

**Figure 1:**
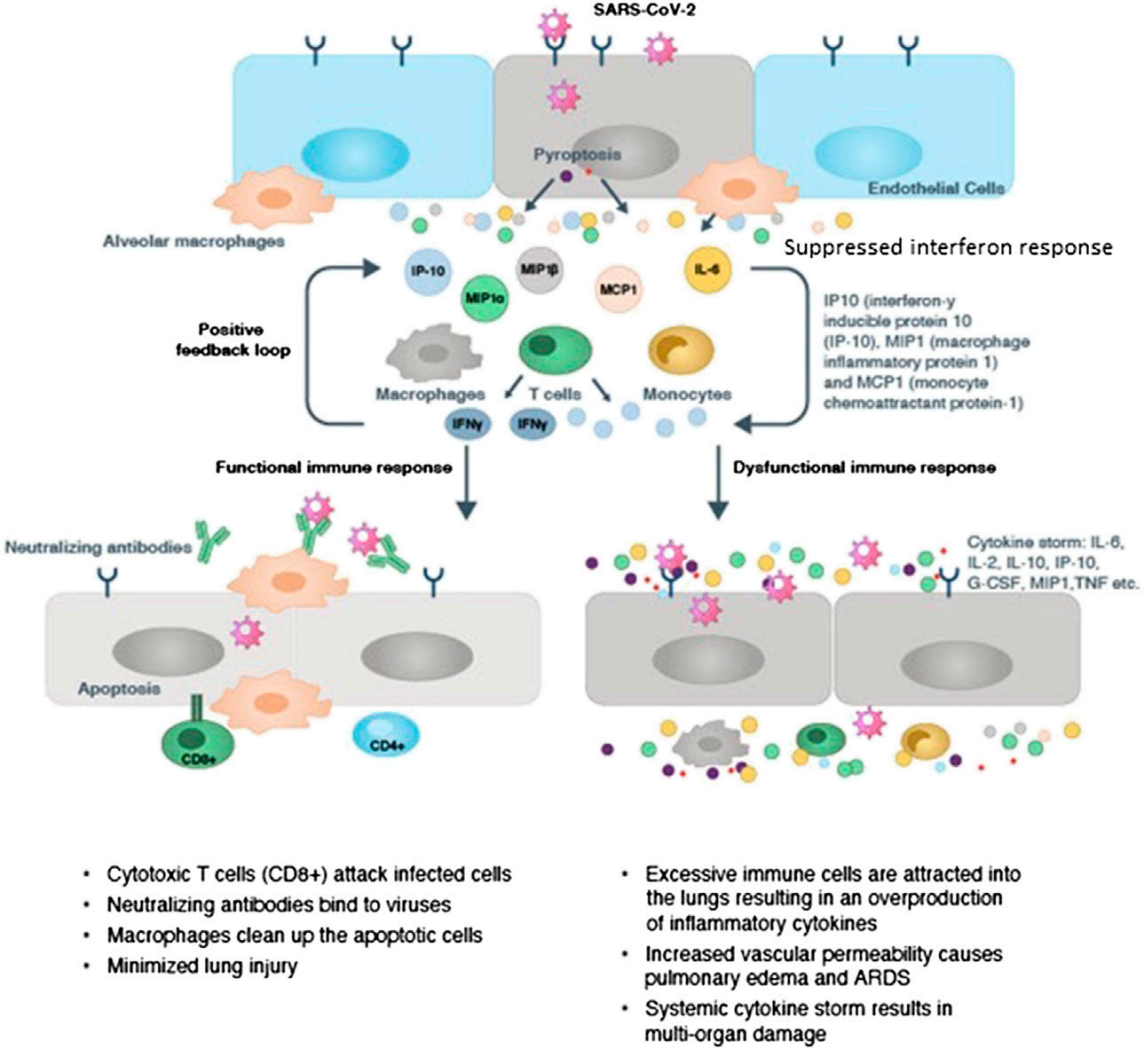
Immunopathology of Cytokine Storm in COVID-19 (CAS, 2020).

Initially, there is a lag in the production of cytokines and chemokines by the innate immune cells in the COVID-19 disease. This is followed by a sudden acute increase in the pro-inflammatory cytokine and chemokine production by the activated macrophages, monocytes and other recruited lymphocytes. The surge in levels of pro-inflammatory cytokines and chemokines augments infiltration of neutrophils, macrophages, monocytes and T-cells to the site of infection, bringing about an intense and violent inflammatory response in the bronchi and alveoli. This leads to disruption of air-blood barrier, endothelial and epithelial cell damage, breakdown of the alveolar-epithelial barrier, diffuse alveolar damage leading to ARDS and pulmonary fibrosis (PF). In addition to the local damage at the site of infection, the cytokine storm also has ripple effects across the body; contributing to viral sepsis, MOF and finally death in critically-ill patients (Schett et al., 2020b; Li et al., 2020c; Lingeswaran et al., 2020; Matthay et al., 2020; Nile et al., 2020; Prompetchara et al., 2020; Ragab et al., 2020; Risitano et al., 2020; Spagnolo et al., 2020; Tay et al., 2020; Vabret et al., 2020). A diagrammatic representation of the cytokine storm observed in COVID-19 is found in Figure 1 (CAS, 2020).

## Immunomodulatory Agents- Targeting the Cytokine Storm in COVID-19

Despite intense scientific study and research, there are no specific drugs or vaccine available for the treatment or prevention of COVID-19. More than 600 clinical trials have been undertaken, with no promising results to date (at the time of submission of this review). The current standard care for COVID-19 being practiced is just symptomatic, palliative and supportive treatment (Catanzaro et al., 2020; Risitano et al., 2020). A diverse spectrum of pharmaceutical agents is being employed in an attempt to treat and efficiently manage COVID-19 infection. Antiviral agents, other chemical agents, monoclonal antibodies and other drugs are part of the current treatment regime. The antiviral drugs in use are lopinavir, ritonavir, remdesivir, oseltamivir, favipiravir, arbidol, ribavirin, ganciclovir and amantadine. Other chemical agents used for treatment are hydroxychloroquine, chloroquine, azithromycin, ivermectin, colchicine, thalidomide and glucocorticoids like methylprednisolone and dexamethasone. Monoclonal antibody tocilizumab, convalescent plasma interferons and intravenous immunoglobulin therapy are some of the other treatment methods in vogue. Additionally, antibiotics like cephalo-sporins, quinolones, carbapenems, linezolid or antifungal agents may be given (Chakraborty et al., 2020a; National Institutes of Health, 2020a; Saha et al., 2020a; Sethi et al., 2020; Tobaiqy et al., 2020).

While the role played by specific antiviral drugs and other pharmaceutical agents that can effectively target SARS-CoV-2 and reduce the infection is significant and cannot be downplayed, the use of immunomodulatory therapy is potentially effective, considering that SARS-CoV-2 drives immune dysfunction and induces hyperinflammation and a cytokine storm in patients (Iannaccone et al., 2020; Shi et al., 2020; Tufan et al., 2020; Zhong et al., 2020). A treatment strategy goes that beyond antiviral therapy alone, using immunomodulatory agents as adjuvants is a holistic therapeutic approach that takes into account the immune response of the host, hence checking the acute immune/ inflammatory response and preventing ARDS and PF (Alijotas-Reig et al., 2020; Liang et al., 2020). The cytokine storm is one prominent feature of the severe immune aberrations observed in COVID-19 patients (Yazdanpanah et al., 2020). Targeting the cytokine storm in order to ameliorate the state of hyperinflammation is proposed to be a novel therapeutic approach in the treatment of COVID-19 patients (Felsenstein et al., 2020; Prompetchara et al., 2020; Roshanravan et al., 2020a; Vabret et al., 2020). Several synthetic drugs, monoclonal antibodies and stem cell therapies have been proposed and are being explored to treat the cytokine storm in COVID-19 and thus reduce the state of hyperinflammation (Alijotas-Reig et al., 2020). Some of the therapeutic approaches being used currently and are also under clinical trials are discussed as follows.

Anti-malarial drugs hydroxychloroquine and chloroquine that are know to decrease Th17-related cytokines; IL-6, IL-17, IL-22, TNFα and IL-1β have been evaluated for efficacy in COVID-19 treatment in several clinical trials (Felsenstein et al., 2020; da Silva et al., 2020; Karres et al., 2020; Meo et al., 2020). Selective cytokine blockade with monoclonal antibodies tocilizumab and sarilumab that are antagonists of IL-6 receptors are being studied in clinical trials in certain countries (Atal and Fatima, 2020; Chakraborty et al., 2020b; Saha et al., 2020b). Calcineurin inhibitors reduce both the calcium-production of IL-2 and the expression of IL-2 receptor thus reducing T-cell activation. Two such inhibitors; cyclosporine and tacrolimus are being considered as potential drug candidates to reduce the cytokine storm syndrome observed in COVID-19 (Mejia et al., 2014; Cure et al., 2020; Rudnicka et al., 2020; National Institutes of Health, 2020b). The IL-1 receptor antagonist anakinra and the anti-IL-1β monoclonal antibody canakinumab are two drug candidates that efficiently treat cytokine storm syndromes observed in other hyper-inflammatory conditions like Still’s disease that are being evaluated for efficacy against the SARS-CoV-2-induced cytokine storm (Huet et al., 2020; Filocamo et al., 2020; National Institutes of Health, 2020c). Infliximab and adalimumab that are anti-TNF antibodies are being considered for modulating the hyperinflammation observed in COVID-19 by specifically targeting TNF (Roshanravan et al., 2020b). Individual studies and clinical trials to evaluate efficacy of low-molecular weight unfractionated heparin in COVID-19 patients with coagulopathy are underway (Tang et al., 2020b; National Institutes of Health, 2020d). Another immunomodulatory treatment molecule being evaluated is the use of intravenous immunoglobulin (IVIG). A clinical trial studying the benefit of administering intravenous immunoglobulin when compared to standard care alone is currently underway (National Institutes of Health, 2020e). Individual studies evaluating the efficacy of high dose of IVIGs as a therapeutic option in deteriorating patients have been undertaken, yielding mixed results (Xie et al., 2020; Cao et al., 2020). Hyperimmune immunoglobulin treatment for COVID-19 is also being explored (Alwis et al., 2020). A clinical trial that compares the efficacy of convalescent plasma to anti-COVID-19 human immunoglobulin in hospitalized patients is currently in progress (National Institutes of Health, 2020f). The JAK/STAT pathway is the main signaling pathway involved in the control of cytokine production (Bagca and Avci, 2020; Seif et al., 2020). Ruxolitinib selectively inhibits Janus kinase (JAK) 1 and 2 and has a modest to marked selectivity against tyrosine kinase (TYK) 2 and JAK 3. Studies and clinical trials are being conducted to evaluate the efficacy of ruxolitinib to target the excess production of cytokines in COVID-19 patients with hyperinflammation and ARDS (La Rosée et al., 2020; National Institutes of Health, 2020g; National Institutes of Health, 2020h). Baricitinib is another inhibitor that binds to JAK 1/2 and inhibits its activation and consequent cytokine release (National Institutes of Health, 2020i). The use of baricitinib for targeting the COVID-19 cytokine storm is being explored in pilot studies and phase 2 and 3 clinical trials (Cantini et al., 2020; National Institutes of Health, 2020j; National Institutes of Health, 2020k). Corticosteroids which are known for their ability to modulate the inflammatory response and have been used in other viral outbreaks are another option for COVID-19 treatment (Russell et al., 2020). Clinical trials testing the prophylactic action, safety and efficacy of dexamethasone and methylprednisolone in reducing the cytokine storm in COVID-19 patients are ongoing (Horby et al., 2020; National Institutes of Health, 2020l; National Institutes of Health, 2020m; Nature, 2020). Statins are a class of drugs that have immunomodulatory and anti-inflammatory properties and are being considered for treating the cytokine storm in COVID-19 (Castiglione et al., 2020; Lee et al., 2020; National Institutes of Health, 2020n). The entry of SARS-CoV-2 into host cells is mediated by hACE2, which is also involved in the virus-induced acute lung injury (Liu et al., 2020). Clinical trials are ongoing, to check the safety and efficacy of recombinant hACE2 (rhACE2) to mediate direct (via restoration of rennin-angiotensin system) and indirect (the chimeric receptor effect) therapeutic effects in SARS-CoV-2 induced inflammation and ARDS (Barone et al., 2020; National Institutes of Health, 2020o; National Institutes of Health, 2020p). The use of multipotent mesenchymal stem cell (MSCs) to inhibit the exaggerated immune response caused by the cytokine storm in COVID-19 patients is also being studied (Rajarshi et al., 2020).

Though the benefits of these synthetic therapeutic immunomodulatory agents cannot be disputed, their use is frequently associated with adverse side effects. Cardiomyopathy, neurological and gastrointestinal side effects are reported with chloroquine and hydroxychloroquine treatment and as COVID-19 patients are more vulnerable because of co-morbidities, there are concerns about safety (Colafrancesco et al., 2020; U.S. Food and Drug Administration, 2020; Gevers et al., 2020). Treatment of COVID-19 patients with IL-6 antagonists with tocilizumab and sarilumab also raises safety concerns as these are known to cause endocrinological, hematological, gastrointestinal and cardiovascular adverse effects (Atal and Fatima, 2020). IL-2 blockers cyclosporine and tacrolimus are known to cause side effects and can be extremely harmful (Cure et al., 2020; Xia et al., 2020). Anakinra and canakinumab, IL-1 blockers have a reasonably good safety profile, but can cause elevation of liver enzyme levels, myopathy and mild leukopenia (Colafrancesco et al., 2020). Anti-TNF treatment adverse effects are reported to be infrequent (Favalli et al., 2020). Severe drug reactions were reported for JAK/STAT inhibitor, ruxolitinib administered to COVID-19 patients; thus prompting the early stoppage of this drug in the study undertaken (Gaspari et al., 2020). There is also a risk of developing serious infections with the use of another oral JAK/STAT inhibitor, baricitinib (Lilly, 2020). Previously conducted clinical trials on statin treatment in ARDS and sepsis patients was met with negative outcomes, thus causing reluctance in considering them as adjuncts for COVID-19 therapy (Lee et al., 2020). In a study of rhACE2 treatment for ARDS patients, hypernatremia and dysphagia were the noted side effects (Barone et al., 2020). In case of improper administration of MSCs, there is a probability of lethal adverse thrombotic complications and reactions necessitating a thorough understanding of this treatment option and application routes (Moll et al., 2020).

## Natural Immunosuppressant Agents- Adjuvants to Target the Cytokine Storm in COVID-19

The discovery of immunomodulatory agents from plants sources with enhanced bioavailability and essentially devoid of toxic side effects is a ray of hope and opens up a novel approach to mitigate the cytokine storm syndrome observed in COVID-19 (Grigore, 2017; Cena and Chieppa, 2020). Several plants and plant-derived bioactive compounds have been studied for their immunomodulatory activity and many have been identified for their immunosuppressive activity in particular (Sagrawat and Khan, 2007; Sahoo and Banik, 2018). These natural immunosuppressive agents provide an alternative therapeutic strategy for the efficient management SARS-CoV-2-associated illness; the cytokine storm and the resulting hyperinflammation. Targeting the cytokine storm with plant-derived immunosuppressants is a very promising strategic treatment method. This is because the individual mediators of the inflammatory cascade; cytokines including IL-1β, IL-6, TNFα and chemokines IP10, MCP1 are neutralized or inhibited, rather than a broad immune suppression that can negatively effect the viral clearance. This specific targeting of cytokines is achieved by the inhibitory action on specific signaling cascades. With their good safety profiles in addition to their capacity to keep a check on the pro-inflammatory cytokine levels all the while without compromising on the ability of the immune system to respond to the SARS-CoV-2; plant-derived immunosuppressant are the need of the hour. Some immunosuppressant agents derived from plants proven to reduce inflammation by downregulating the levels of pro-inflammatory cytokines that can be used as adjuvants in COVID-19 treatment are discussed as follows.

### Andrographolide

Andrographolide is a labdane diterpenoid; 4-hydroxy-3-[2-[6-hydroxy-5-(hydroxymethyl)-5,8a-dimethyl-2-methylidene-3,4,4a,6,7,8-hexahydro-1H-naphthalen-1-yl]ethylidene]oxolan-2-one. It is isolated from the medicinal plant Andrographis paniculata (Brum.f.) Nees and has a wide range of therapeutic properties; anti-inflammatory, anti-allergic, anti-platelet aggregation, antineoplastic, anti-HIV and hepatoprotective activity (Jayakumar et al., 2013; National Center for Biotechnology Information, 2020a). Andrographolie is also a potent immunomodulator; known to significantly stimulate the immune response, regulate the production of NK cells and cytokines and stimulate the production of cytotoxic T-lymphocytes (Varma et al., 2011). Andrographolide efficiently brought about a dose-dependent reduction in the levels of inflammatory cytokines TNFα, IL-12, IL-1β, IL-6, IL-18 in LPS/ IL-4-activated murine macrophages (Wang et al., 2010). Andrographolide treatment suppresses inflammatory mediators IL-1β, TNFα, prostaglandin E2 (PGE2), NADPH oxidase 2 (NOX2) and inducible nitric oxide synthase (iNOS) in ischemic brain tissues after pMCAO stimulation (Lu et al., 2019). In LPS-stimulated RAW264.7 cells, andrographolide treatment resulted in a similar dose-dependent reduction in levels of pro-inflammatory cytokines TNFα, IL-1β and IL-6 and their corresponding mRNA expression levels. This reduction in pro-inflammatory cytokine secretion is because andrographolide suppresses LPS-induced NF-κB and MAPK pathways; andrographolide reduced the levels of p65 and IkBα phosphorylation in the NF-κB pathway and the levels of *p*-JNK, *p*-ERK1/2 and p-p38 in MAPK pathway (Li et al., 2017). Andrographolide derivatives were found to inhibit TNFα/ NF-κB and TLR4/ NF-κ signaling pathways by inhibiting the nuclear translocation of the NF-κB p65 subunit and attenuating the phosphorylation of p65 and IκBα, thus decreasing the levels of serum pro-inflammatory cytokines and chemokines (Nie et al., 2017). In a study, oral administration of andrographolide significantly attenuated mouse cortical chemokine levels from the C-C (CCL2, CCL5) and C-X-C (CXCL1, CXCL2, CXCL10) subfamilies. Andrographolide also abrogated LPS-induced chemokines (CCL2, CCL5, CXCL1, CXCL5, CX3CL1) and TNFα in astrocytes (Wong et al., 2016). In a study carried out using Jurkat cells stimulated with phorbol myristate acetate and ionomycin (PMA/ionomycin), andrographolide was found to reduce the production of IL-2 and reduce the activity of NF-κB. It also brought about a decrease in the ERK1 and ERK5 phosphorylation induced by anti-CD3 or PMA/ionomycin (Carretta et al., 2009). Andrographolide significantly reduced the production of INFγ and partially inhibited IL-2 production in murine T-cells stimulated with concanavalin A. This reduced INFγ production was associated with a significant decrease in the ERK1/2 phosphorylation on treating the cells with andrographolide. In the same study, andrographolide was also found to reduce hydrocortisone/PMA-induced apoptosis in thymocytes (Burgos et al., 2005). Docking studies carried out revealed that andrographolide is also a potent inhibitor of the main protease of SARS-CoV-2 (Mpro). Moreover, andrographolide is safe and does not interfere with the metabolism of other therapeutic drugs (Enmozhi et al., 2020). This dual beneficial role of andrographolide; as a potent immunosuppressive agent to alleviate the abnormal cytokine and chemokine production and as a potential inhibitor of SARS-CoV-2 by targeting the main protease make andrographolide a promising natural agent to be considered for COVID-19 targeted therapy (Banerjee et al., 2020).

### Allicin

Allicin is a thiosulphate; 3-prop-2-enylsulfinylsulfanylprop-1-ene which is a constituent of garlic oil responsible for the typical smell and taste associated with freshly cut garlic (Allium sativum L.). Allicin is also found in garden onion (Allium cepa L.) and in other species in the family Alliaceae (Borlinghaus et al., 2014; National Center for Biotechnology Information, 2020b). Allicin is known to possess antioxidant, antimicrobial, antiviral, anti-inflammatory, anti-tumor and anti-diabetic properties (Batiha et al., 2020). In a study undertaken, allicin brought about a marked inhibition in the spontaneous and TNF-induced secretion of IL-1β, CXCL8 and IP10 in a dose-dependent manner in two different cell lines of intestinal epithelial cells. The mRNA levels of the IL-1β cytokine and CXCL8 chemokine are also reduced. This reduction in cytokine production is because of the effect of allicin on the NF-κB pathway; it suppresses the degradation of IκB (Lang et al., 2004). Allicin treatment to BALB/c mice post Plasmodium yoelii infection resulted in improved defense and survival due to an increase in the production of IFNγ and expansion of CD4^+^ T-cells. Allicin supplementation along with tamoxifen treatment to Ehrlich ascites carcinoma (EAC) cells resulted in marked decrease in TNFα levels, showing its beneficial role as an adjuvant (Arreola et al., 2015). In clinical trials evaluating the safety of allicin treatment, no adverse effects were reported (Sharifi-Rad et al., 2019). In HT-29 and Caco-2 cells stimulated with TNFα, allicin brought about a marked inhibition in the secretion of IL-1β, IP10, CXCL8 and MIG in a dose-dependent manner. RNA protection assay revealed that allicin reduced the expression of IL-1β and CXCL8 mRNA levels (Lang et al., 2004). Allicin was also responsible for suppressing the degradation of IkappaB. In LPS-treated blood samples, allicin drastically reduced the release of IL-10 (Keiss et al., 2003). Allicin successfully attenuated the LPS-induced inflammatory responses in a study with cultured human umbilical vein endothelial cells (HUVECs). Significant decrease in the levels of TNFα, CXCL8 and NFκB activity were observed on treatment with allicin. Additionally, treatment with allicin also reduced LPS-induced apoptosis in the cultured HUVECs (Zhang et al., 2017). With its ability to alter NF-κB signaling for reduction in cytokine secretion potentially targeting the cytokine storm, improve defense by increasing the IFNγ production for increased antiviral defense and improve expansion of CD4^+^ T-cells thus targeting the lymphocytopenia, allicin is a promising natural immunomodulatory adjuvant that can be used in conjunction with antiviral therapy in COVID-19 patients. However, its safe use in COVID-19 patients necessitates clinical trials to evaluate safety profile and pharmacokinetics (with antiviral agents in use) in these patients.

### Colchicine

Colchicine is a secondary metabolite (alkaloid) extracted from Colchicum autumnale L. and Gloriosa superb L. (Varsha et al., 2017). Chemically, colchicine is N-[(7S)-1,2,3,10-tetramethoxy-9-oxo-6,7-dihydro-5H-benzo[a]heptalen-7-yl]acetamide (National Center for Biotechnology Information, 2020c). It is known to possess anti-inflammatory and immunomodulatory properties and is used in the treatment of gout, FMF and Beçhet’s syndrome. Colchicine is known to inhibit the production of IL-1 (Soy et al., 2020). Microtubules form an important part of the cytoskeleton, which is involved in cellular processes like secretion of cytokines and chemokines and cell migration. Colchicine blocks the polymerization of microtubule by binding to the *ß*-tubulin subunit with high affinity, thus preventing its assembly and affecting the process of cytokine and chemokine secretion and the migration of inflammatory cells like monocytes and neutrophils. Colchicine also disrupts the activation of inflammasome, suppressing activation of caspase-1 and subsequent release of IL-1β and IL-18 (Montealegre-Gómez et al., 2020). In an experimental study on patients with acute coronary syndromes, colchicine was found to bring a significant reduction in the levels of inflammatory cytokines IL-1β, IL-6 and IL-18 (Martínez et al., 2015). Colchicine was also found to bring about the inhibition of the assembly of the NLRP3 inflammasome thus resulting in the inhibition of the expression of pro-inflammatory cytokines including IL-1β. Given the increased NLRP3 inflammasome activation that contributes to hypercytokinemia observed in SARS-CoV-2 patients, colchicine is definitely an agent of interest to suppressing the NLRP3 inflammasome and thus ameliorate the state of hypercytokinemia observed (Papadopoulos et al., 2020; Ribeiro et al., 2020; Shah, 2020; Vitiello et al., 2020). Individual studies have been undertaken to evaluate the efficacy of cholchicine treatment for COVID-19. Colchicine was observed to bring about a reduction in the cytokine levels, activation of neutrophils and macrophages and inflammasome in SARS-CoV-2 patients. Adverse effects in patients were minimal, mild diarrhea was only effect reported in few patients (Della-Torre et al., 2020; Montealegre-Gómez et al., 2020). Clinical trials are underway to consider the clinical utility, safety and efficacy of colchicine-adjuvant therapy for COVID-19 (Soy et al., 2020).

### Curcumin

Curcumin (1E,6E)-1,7-bis(4-hydroxy-3-methoxyphenyl)hepta-1,6-diene-3,5-dione) is a polyphenol pigment derived from the perennial plant Curcuma longa L., commonly known as the turmeric spice. It is a natural antioxidant, possesses anti-inflammatory, neuroprotective and hepatoprotective activity, and also inhibits tumor cell proliferation. Curcumin is also an immunomodulator (Hewlings and Douglas, 2017; National Center for Biotechnology Information, 2020d). The ability of curcumin to inhibit the production and release of different pro-inflammatory cytokines and chemokines IL-1β, IL-2, IL-6, IL-12, TNFα, CXCL8, IP10, MCP1, MIP1α and IFNγ has been proved in several studies. This immunosuppressive action of curcumin is mediated by its effect on different signaling pathways. Curcumin regulates the NF-κB pathways at multiple stages; inhibiting activation of IKKβ, blocking cytokine-mediated NF-κB activation by inhibiting IκBα degradation, blocking NF-κB signaling by activating AMPK, disturbing the NF-κB pathway by acting on p65 and as a consequence downregulating transcription of cytokine genes. On the other hand, curcumin positively regulates anti-inflammatory cytokines, particularly IL-10. IL-10 reduces levels of TNFα, and ICAM 1. Curcumin was found to increase the expression, production and activity of IL-10 (Jain et al., 2009; Sordillo and Helson., 2015; Shimizu et al., 2019; Liu et al., 2020). A study on the effect of curcumin on the pro-inflammatory cytokine IL-18 in LPS-stimulated murine macrophage-like cells RAW264.7 revealed that curcumin significantly inhibited the production of IL-18 (Yadav et al., 2015). In another study on monocyte culture exposed to pre-eclamptic plasma, curcumin caused a significant reduction in the levels of pro-inflammatory cytokines IL-1α, IL-6 and TNFα and also brought about a decrease in the level of nuclear NF-κB p50 (Rahardjo et al., 2014). In Iran, a randomized, double-blind, placebo-controlled study was carried out on COVID-19 patients form the Imam Reza Hospital of Tabriz University of Medical Sciences was carried out. This study aimed to evaluate the efficacy of nano-curcumin (dose- 160 mg of in four 40 mg capsules daily for 14 days) in modulating the levels of inflammatory cytokines IL-1β, IL-6, TNFα and IL-18 in 40 patients. Decreased expression and secretion of IL-1β and IL-6 was observed in the COVID-19 patients. However, there was no positive effect on the IL-18 mRNA expression and TNFα concentration with the treatment of nano-curcumin (Babaei et al., 2020; Valizadeh et al., 2020; Zahedipour et al., 2020). Being a natural spice extract, curcumin is safe and has low toxicity. All these properties motivate further clinical investigations for the use of curcumin as an immunomodulatory adjuvant to mitigate the cytokine storm, hyperinflammation and ARDS observed as a result of SARS-CoV-2 infection (Sordillo and Helson., 2015; Liu and Ying, 2020; Liu et al., 2020).

### Eugenol

An aromatic oil, eugenol is 2-methoxy-4-prop-2-enylphenol. Although eugenol is also called clove oil as it is the main component of clove buds (Syzygium aromaticum (L.) Merr. and L.M. Perry), it is found many other plants like cinnamon bark and leaves, tulsi leaves, turmeric, pepper, ginger and organic herbs like oregano, thyme, basil, bay, marjoram, mace and nutmeg (Khalil et al., 2017; National Center for Biotechnology Information, 2020e). Eugenol is known to possess multiple beneficial pharmacological properties. It is a potent antioxidant, analgesic, antimicrobial, anticonvulsant and anticancer agent. Additionally it possesses anti-inflammatory and anti-viral properties and is an immunomodulatory agent (Pramod et al., 2010; Dibazar et al., 2015). In a study, eugenol inhibited the production of IL-6 and IL-10 *in vitro* (Bachiega et al., 2012). Administration of eugenol prevented increase in IL-4 and IL-5, reduced NF-κB signaling pathways and ultimately protected lung tissue from OVA-induced eosinophilia. In another study, eugenol caused the downregulation of pro-inflammatory cytokines IL-6 and TNFα; reduced the expression of NF-κB signaling, thus reducing leukocyte recruitment and inflammation in the lung tissue. Eugenol also reduced the TNFα and IL-1β as well as the NF-κB, ERK1/2, and p38 MAPK signaling pathways in LPS-induced macrophages (Barboza et al., 2018). When used in low doses, eugenol has few adverse effects; local irritation and rare allergic reactions. In case of accidental overdose, tissue injury and damage to liver and kidney can result (National Institute of Diabetes and Digestive and Kidney Diseases, 2020). Treatment with polymeric nanocarriers of eugenol was found to bring about a significant reduction in the TPA-induced IL-6 levels in Swiss mice (de Araújo Lopes et al., 2018). In a LPS-induced inflammatory model in porcine intestinal epithelial cells, eugenol enervated the inflammatory response by reducing both, the CXCL8 and TNFα mRNA levels significantly (Hui et al., 2020). Another study carried out on LPS-challenged macrophages revealed that eugenol suppressed the production of IL-6 and IL-10. However, there was no effect observed on the production of IL-1β (Bachiega et al., 2012). Mouse peritoneal macrophages were subject to activation with a bacterial LPS in order to evaluate the effect of eugenol on pro-inflammatory mediator genes NF-κB1 and TNFα. Since LPS did not induce the expression of NF-κB1, the effect of eugenol could not be ascertained. In case of TNFα, hypoexpression of was observed on treatment with eugenol in LPS-activated cells (Porto Mde et al., 2014). The effect of eugenol treatment on the two important inflammatory markers IL-6 and TNFα was examined in adult male Wistar rats. The levels of both IL-6 and TNFα were elevated post thioacetamide-induced hepatic injury. Pretreatment with eugenol brought about a significant decrease in the levels of these inflammatory markers (Yogalakshmi et al., 2010). With the good safety profile of eugenol and its ability to downregulate key pro-inflammatory cytokines like IL-6 and TNFα consequently reducing leukocyte recruitment in lung tissue, it can be a suitable natural immunosuppressant that can be used as an adjuvant along with antiviral agents to suppress the hypercytokinemia and hyperinflammation observed in COVID-19.

### Gallic Acid

Gallic acid is a phenolic secondary metabolite found in abundance in plants; in the bark, wood, leaf, fruit, root and seeds. Chemically, it is 3,4,5-trihydroxybenzonic acid (Fitzpatrick and Woldermariam, 2017). It is a natural antioxidant that also has anticancer, antifungal, antimicrobial and anti-inflammatory properties. Studies revealed that gallic acid suppresses the levels of pro-inflammatory cytokines IL-1, IL-6, IL-12, IL-17, IL-23, TGFβ, TNFα and chemokines CCL2 and CCL7 in TNBS-induced ulcerative colitis and RA FLS. This anti-inflammatory activity of gallic acid is exerted by inhibiting of NF-κB pathway (Huang et al., 2019; Zhu et al., 2019). Another study revealed that gallic acid selectively suppressed Th2 cytokines IL-4 and IL-5 but did not suppress the Th1 cytokine IFNγ in anti CD3-stimulated spleen cells (Kato et al., 2001). Treatment with gallic acid was found to suppress the activity of NF-κB and inhibited pro-inflammatory cytokine release in high glucose-induced human monocytes (THP-1 cells) (Lee et al., 2015). The inhibitory effect of gallic acid on the production of pro-inflammatory cytokines TNFα and IL-6 was evaluated in human mast cells (HMC-1). HMC-1 cells were stimulated with PMA plus A23187 which caused the secretion of both TNFα and IL-6. Gallic acid treatment significantly blocked the secretion of both these cytokines in the stimulated HMC-1 cells (Kim et al., 2006). Gallic acid treatment was also found to successfully inhibit the PMA plus A23187-induced degradation of IκBα and the nuclear translocation of p65 NF-κB. The inflammatory regulatory effect of gallic acid was assessed in LPS-induced endometriosis cells. Gallic acid was responsible for a significant decrease in the expression of NF-κB and in the secretion profile of pro-inflammatory cytokine IL-6 (Bustami et al., 2018). The effect of polygallic acid, enzymatically produced from gallic acid on the secretion of pro-inflammatory cytokines IL-6, TNFα and IL-1β was evaluated in human monocytes exposed to PMA. A substantial decrease in the production of all three cytokines under study was observed on treatment with polygallic acid (Zamudio-Cuevas et al., 2020). This suggests that gallic acid can be used as an immunosuppressive adjuvant, to selectively suppress pro-inflammatory cytokines thus targeting the cytokine release syndrome observed in SARS-CoV-2 patients without negatively affecting the ability of the host to produce interferons.

### Gingerol

A constituent of fresh ginger (Zingiber officinale Roscoe), gingerol is a beta-hydroxy ketone that is 5-hydroxydecan-3-one substituted by a 4-hydroxy-3-methoxyphenyl moiety at position 1 (5S)-5-hydroxy-1-(4-hydroxy-3-methoxyphenyl)decan-3-one (National Center for Biotechnology Information, 2020f). Gingerol is also found in the rhizomes of several members of the Zingiber species. Gingerol possesses antioxidant, anti-inflammatory, anti-metastatic, anti-angiogenic, anti-diabetic, analgesic and antipyretic activity. Additionally, gingerol is also known to possess immunomodulatory properties and anti-allergic activity (Sharifi-Rad et al., 2017). 6-gingerol was found to exert an inhibitory effect on the production of pro-inflammatory cytokines IL-1β, IL-12, TNFα in LPS-stimulated macrophages, without interfering with their antigen presentation ability (Tripathi et al., 2007). In another study, treatment with an effective dose of 50 µM of 6-gingerol was found to reduce the V. cholerae-infection triggered levels of cytokines IL-1α, IL-1β, IL-6 and chemokine CXCL8. The respective mRNA levels were also reduced. This reduction in the levels of pro-inflammatory cytokines was attributed to the down-regulation of NF-κB pathway because of increase in phosphorylated IKBα and downregulation of p65 (Saha et al., 2016). Another study reported that 6-gingerol reduced IL-1β-induced inflammation by reducing the mRNA titers of IL-6 and CXCL8, reducing the COX2 over-expression and the NF-κB activity (Li et al., 2013). In a DSS-induced mouse colitis model, 6-gingerol treatment was found to successfully suppress the DSS-elevated production of pro-inflammatory cytokines IL-1β, IL-12 and TNFα. An *in vitro* study on DSS-treated Caco-2 cells subject to 6-gingerol therapy revealed AMPK activation following treatment; suggesting that the anti-inflammatory effect of 6-gingerol could be exerted through AMPK activation (Chang et al., 2015). The production of pro-inflammatory cytokines TNFα, IL-1β and IL-12 in LPS stimulated murine peritoneal macrophage was inhibited on treating with 6-gingerol (Tripathi et al., 2007). In another study carried out on C6 astroglioma cells treated with LPS, treatment with 6-gingerol was found to attenuate the production of pro-inflammatory cytokines in a dose dependent manner. Additionally, in the *in vivo* studies, 6-gingerol also inhibited the LPS-induced increase of TNFα levels in the rat brain (Zhang et al., 2018). The effect of 6-gingerol on cytokine production was evaluated in a DSS-induced colitis mouse model. 6-gingerol inhibited the DSS-stimulated rise in the serum levels of cytokines IL-6 and IL-17 and their respective mRNA levels. Moreover, it also inhibited the DSS-induced decrease in the serum levels and mRNA levels of the anti-inflammatory cytokine. IL-10 (Sheng et al., 2020). Gingerol is a potential natural immunomodulatory agent that may prove to be useful in regulation of the cytokine storm observed in COVID-19 when used in conjunction with standard antiviral therapy.

### Luteolin

A naturally occurring flavonoid, luteolin is 2-(3,4-dihydroxyphenyl)-5,7-dihydroxychromen-4-one. It is found in many vegetables (celery, parsley, broccoli, carrots, peppers, and cabbages); fruits (apple skins); flowers (chrysanthemum) and in medicinal herbs. It is a potent antioxidant, inhibits tumor cell proliferation and suppresses metastasis. It is also an anti-inflammatory agent and an immune system modulator (Lin et al., 2008; National Center for Biotechnology Information, 2020g). Luteolin exhibits a concentration-dependent inhibition of the production of TNFα and IL-1β in LPS/IFN-induced primary microglia and BV-2 microglial cells. This inhibitory effect results from an inhibition on NF-κB, STAT1 and IRF1; all essentially required for the transcription of pro-inflammatory genes (Kao et al., 2011). In another study, a significant inhibition of TNFα, IL-6, CXCL8, GM-CS and suppression of NF-κB activation was observed with luteolin treatment on PMA plus A23187-induced HMC-1 cells (Kang et al., 2010). In human monocytes under hyperglycemic condition, luteolin brought about a significant reduction in the release of IL-6 and TNFα by suppressing NF-κB activity (Kim et al., 2014). In a study with human whole blood incubated with LPS, luteolin effectively inhibited the production of IL-1β, IL-6, TNFα and IFNγ (Ribeiro et al., 2010). Primary murine microglia and BV-2 microglial cells stimulated with LPS exhibited significant increase in levels of inflammatory cytokine, IL-6. Pretreatment with luteolin was found to attenuate this increase in IL-6; both at mRNA and protein level. While luteolin was found to bring about a marked reduction in the binding activity of AP1 transcription factor and inhibit JNK phosphorylation, it had no significant effect on the LPS-induced increase in NF-κB DNA binding activity nor the LPS-induced IκBα degradation. Furthermore, the *in vivo* studies on mice treated with luteolin revealed that it successfully reduced the plasma levels of IL-6 and the mRNA levels of IL-6 in the hippocampus, but not in the cortex or cerebellum. This inhibitory activity of luteolin on the LPS-induced production of IL-6 can be attributed to its ability to inhibit the both JNK signaling pathway and the activation of AP1 in the microglia (Jang et al., 2008). Luteolin was also found to significantly reduce the serum levels of pro-inflammatory cytokines IL-1β, IL-6 and TNFα in MSU induced-inflammation in rats (Lodhi et al., 2019). Luteolin effectively inhibited the production of TNFα and IL-6 in LPS/INFγ stimulated RAW264.7 cells. NF-κB expression was also suppressed in a dose-dependent manner, following luteolin treatment (Lee et al., 2016). Luteolin pretreatment was found to bring a marked decrease in the mRNA expression and the release of IL-6, IL-8 and VEGF in a dose dependent manner in human HaCaT and primary keratinocytes on TNF stimulation. The TNF-induced phosphorylation, nuclear translocation and DNA binding of NF-κB was significantly brought down by treatment with luteolin. Furthermore, the TNF-induced mRNA expression of the NFKB1 and RELA genes which encode the two NF-κB subunits (NF-κB p50 and NF-κB p65, respectively) was also reduced by luteolin treatment, suggesting that luteolin reduces pro-inflammatory cytokine production by decreasing NF-κB induction (Weng et al., 2014). Luteolin also has broad anti-viral activity, inhibits mast cells and potentially inhibits SARS-CoV-2 main protease (3CLpro) as revealed in recent in docking studies (Theoharides, 2020). Taken together with its immunomodulatory properties, luteolin is an important natural agent for consideration as a potential therapeutic that can be further evaluated as an adjuvant to ameliorate the cytokine storm observed in COVID-19 and to potentially inhibit SARS-CoV-2 infection. Further clinical trails in this regard to ascertain the safety, efficacy and dosage for COVID-19 treatment needs to be conducted.

### Melatonin

Melatonin is a bioactive compound reported to be found in numerous plants species. Chemically, it is a tryptophan-derived substituted indolamine; N-acetyl-5-methoxytryptamine. It is a universal amphiphilic antioxidant and an anti-inflammatory molecule (Salehi et al., 2019). Melatonin is found in the roots of Huang-qin (*Scutellaria baicalensis Georgi*.), curcuma (*Curcuma aeruginosa Roxb*.); leaves and flowers of St. John’s wort (*Hypericum perforatum L*.); leaves of *Tanacetum parthenium* (L.) Sch. Bip., black pepper (*Piper nigrum* L.); seeds of corn (*Zea mays* L.), rice (*Oryza sativa* L.), black mustard (*Brassica nigra* (L.) K.Koch), white mustard (*Brassica hirta Moench*), wolf berry (*Lycium barbarum* L.), fennel (*Foeniculum vulgare Mill*.), sunflower (*Helianthus annuus L*.), fenugreek (*Trigonella foenum-graecum* L.), barley (*Hordeum vulgare L*.), almonds (*Prunus amygdalus Batsch*), coriander (*Coriandrum sativum L*.), celery (*Apium graveolens* L.), anise (*Pimpinella anisum* L.), poppy (*Papaver somniferum* L.), flax (*Linum usitatissimum* L.) and in the bean of coffee (Coffea canephora Pierre. Ex A.Froehner, *Coffea arabica* L.). The presence of melatonin has been reported in many plant species belonging to the families Rosaceae, Vitaceae, Poaceae, Apiaceae and Brassicaceae (Nawaz et al., 2016). Melatonin has been reported to possess indirect anti-viral activity due to its anti-inflammatory activity. It down-regulates the production and release of pro-inflammatory cytokines, inflammation and cell recruitment. This down-regulation and consequent anti-inflammatory activity of melatonin is because it suppresses the activation of NF-κB, a key transcription factor involved in the production of pro-inflammatory cytokines. In addition, studies revealed that melatonin is able to cause significant decrease in serum levels of IL-6, TNFα, IL-1β (Tarocco et al., 2019; Zhang et al., 2020b). Melatonin efficiently suppressed the production of IL-8 in acrolein-induced human pulmonary fibroblasts (Kim et al., 2012). Treatment with melatonin was found to significantly suppress pro-inflammatory cytokine production and inhibit NLRP3 inflammasome activation in a study carried out in cadmium–induced liver injury in male C57BL/6 mice (Cao et al., 2017). In another study carried out on BV2 murine microglial cells, the LPS-induced production of chemokines MCP1, CCL5 and CCL9 mRNA expression was significantly inhibited by melatonin treatment. Melatonin also inhibited the AkT phosphorylation and NF-κB activation induced by LPS exposure. Moreover, LPS-induced STAT1/3 phosphorylation and interferon-gamma activated sequence (GAS)-driven transcriptional activity was also inhibited as a result of melatonin treatment. This suggests that the anti-inflammatory activity of melatonin is mediated by inhibiting the NF-κB and STAT/GAS activation in LPS-stimulated BV2 murine microglial cell line (Min et al., 2012). A recent mechanistic analysis suggested that supplementation with melatonin can efficiently downregulated the cytokine storm in COVID-19 by reversing aerobic glycolysis in immune cells (Reiter et al., 2020). Short-term usage of melatonin is safe; melatonin has a high safety profile. Infrequently occurring side-effects reported are only occasional dizziness, headache, nausea and sleepiness. These findings support the use of melatonin as an immunosuppressive adjuvant to reduce the cytokine storm syndrome observed in COVID-19 (Zhang et al., 2020b).

### Morphine and Codeine

An opiate alkaloid obtained from the plant *Papaver somniferum L*., morphine (4R,4aR,7S,7aR,12bS)-3-methyl-2,4,4a,7,7a,13-hexahydro-1H-4,12-methanobenzofuro[3,2-e]isoquinoline-7,9-diol) binds to and activates the *δ*, *µ*, *κ* opiate receptors involved in controlling different brain functions. Morphine is an analgesic, brings about sedation, respiratory depression and gastrointestinal system smooth muscle contraction (National Center for Biotechnology Information, 2020h). The immunosuppressive activity of morphine has been known in clinical medicine for over hundred years (Dinda et al., 2005). Several studies have established the immunosuppressive activity of morphine with respective to cytokine production. *In vivo* studies have revealed that morphine inhibits the production of TNFα, IFNγ, MCP 1, IL-12 and IL-6 (Clark et al., 2007; Fukada et al., 2012; Cruz et al., 2017). Morphine has the ability to reduce the production of TNFα, IL-1 and IL-6 (Chang et al., 2011). Another study evaluated the effect of morphine on the intraperitoneal release of TNFα and MCP1 in Swiss–Webster, C57BL/6J, mast cell deficient Kit Wsh/Wsh (W-sh) and mast cell reconstituted (W-sh-rec) mice. While morphine inhibited the LPS-induced TNFα release, it had no significant effect on the MCP1 release in the intraperitoneal cavity (Madera-Salcedo et al., 2011). Morphine treatment resulted in significant reduction in the levels of TNFα, IL-1, IL-2 and MIP2 in the bronchoalveolar lavage fluids and in lung tissue in CB6F1 male mice. Morphine treatment also inhibited transcription factor NF-κB in lung resident cells (Wang et al., 2005). Codeine (4R,4aR,7S,7aR,12bS)-9-methoxy-3-methyl-2,4,4a,7,7a,13-hexahydro-1H-4,12-methanobenzofuro[3,2-e]isoquinolin-7-ol) is a naturally occurring phenanthrene alkaloid and an opioid agonist also obtained from P. somniferum L., also known for its analgesic, anti-diarrheal and antitussive properties (National Center for Biotechnology Information, 2020i). Treatment with codeine was found to significantly suppress the production of IL-2 in ConA-stimulated splenocytes extracted from male Swiss mice (Sacerdote et al., 1997). Morphine and codeine bind directly to ACE2 with high affinity, as revealed by docking studies. This can hypothetically reduced receptor-mediated cytokine release. This makes morphine and codeine candidate immunosuppressant adjuvants for regulating the cytokine storm observed in COVID-19 (Roshanravan et al., 2020b). However, further studies need to be conducted to study the use of morphine or codeine for COVID-19, given that the timing of morphine administration greatly effects its influence on cytokine production; late administration increases cytokine production and that codeine was found to induce the production of cytokines and chemokines by mast cells *in vitro* (Sheen et al., 2007; Fukada et al., 2016).

### Nicotine

Nicotine is a plant alkaloid found in the tobacco plant (*Nicotiana tabacum L*.). Chemically, it is 3-(1-methylpyrrolidin-2-yl) pyridine. It is an immunomodulator, a peripheral nervous system drug and a psychotropic drug (National Center for Biotechnology Information, 2020j). Nicotine is can also be found in some members of the families Asclepiadaceae, Crassulaceae, Solanaceae (Dawson et al., 1960). It is used clinically in the treatment of ulcerative colitis to counteract inflammation. As it is a cholinergic agonist, nicotine is an inhibitor of pro-inflammatory cytokines acting through the cholinergic anti-inflammatory pathway via a7-nicotinic acetylcholine receptor (a7-nAChRs). Nicotine inhibits TNF, IL- 1 and IL-6 production. The underlying mechanism of action involves the activation of a7-nAChRs on inflammatory cells like macrophages and neutrophils that induces the suppression of the NF-κB activation and consequently suppression of the secretion of pro-inflammatory cytokines and chemokines from inflammatory cells. Nicotine brought about an inhibition in the secretion of IFNγ, TNFα, IL-1β and IL-2 but had no effect on the production of IL-6 in as study carried out on PBMC triggered by HT-29 colon carcinoma cells. However, nicotine treatment in the in PBMC triggered by RKO colon carcinoma cells showed no suppressive effect on the cytokine production (Djaldetti and Bessler, 2017). Nicotine inhibits the LPS-induced production of TNFα and NF-κB in human macrophages and splenocytes. This inhibitory effect is attributed to the ability of nicotine to activate JAK2 and STAT3 and is mediated by tristetrapolin (TTP) expression in macrophages (Lakhan and Kirchgessner, 2011). Studies have showed that nicotine has the ability to inhibit the production of IL-1, IL-6, IL-12, INFγ, MIP1, TNFα. Nicotine inhibits the transcriptional activity of NF-κB by suppressing the phosphorylation of I-κB (Cloëz-Tayarani and Changeux, 2007; Piao et al., 2009). Nicotine is also an accessible, approved treatment. Hence, it is hypothesized that nicotine is a suitable adjuvant that can ameliorate the cytokine storm in COVID-19 and can likely reduce the rising mortality in the short term (Farsalinos et al., 2020; Gonzalez-Rubio et al., 2020).

### Piperine

Piperine is an alkaloid obtained from the plant Piper nigrum L. and other plants from the family Piperaceae. Chemically, it is N-acylpiperidine that is piperidine substituted by a (1E,3E)-1-(1,3-benzodioxol-5-yl)-5-oxopenta-1,3-dien-5-yl group at the nitrogen atom (2E,4E)-5-(1,3-benzodioxol-5-yl)-1-piperidin-1-ylpenta-2,4-dien-1-one (National Center for Biotechnology Information, 2020k). Piperine is known to possess numerous therapeutic effects; antioxidant, anti-inflammatory, antimicrobial, anti-metastatic and hepatoprotective activities, to name a few. It is also an immunomodulator and is known for its bioavailability enhancement property of therapeutic drugs (Gorgani et al., 2017). Piperine was reported to inhibit the production of Th2 cytokines IL-4 and IL-5 in an ovalbumin-induced asthma model (Kim and Lee, 2009). Another study reported that piperine inhibits LPS-induced expression and production of pro-inflammatory cytokines IL-1β, IL-6, IL-10 and TNFα by inhibiting NF-κB activation (Dzoyem et al., 2017). Pipeine also protects macrophages from pyroptosis and suppresses the release of IL-1β by suppressing ATP-induced AMPK activation in murine macrophages (Liang et al., 2016). In another study carried out using human PBMC, piperine was found to inhibit the production of IL-2 and IFNγ in a dose-dependent manner by suppressing the IL-2 and IFNγ mRNA expression, as revealed by ELISA assay and RT-PCR data (Chuchawankul et al., 2012). Piperine was found to selectively suppress the expression of S. aureus-induced pro-inflammatory cytokines IL-1β, IL-6 and TNFα and increase the expression of the anti-inflammatory cytokine IL-10 by inhibiting the NF-κB and MAPK pathways in an experimental study in mice (Zhai et al., 2016). The IL-6 expression by IL-1β stimulated fibroblast-like synoviocytes derived form patients with rheumatoid arthritis was successfully inhibited by piperine treatment. Additionally, piperine could inhibit the migration of activator protein 1 (AP-1), but not nuclear factor (NF)κB into the nucleus (Bang et al., 2009). Piperine was found to bring about a reversal in the LPS-induced increase of pro-inflammatory cytokines IL-1β, IL-6 and TNFα production in a study carried out on human colonic epithelial cells. This attenuation of cytokine release was attributed to the ability of piperine to activate IκBα and consequently suppress NF-κB expression (Buagaew et al., 2020). Significant reduction in the levels of IL-1β, IL-6, TNFα and GM-CSF in B16F-10 melanoma cells was observed on treatment with piperine. Additionally, piperine treatment also reduced the nuclear translocation of p65, p50, c-Rel subunits of NF-kappaB and other transcription factors such as ATF-2, c-Fos and CREB. Piperine inhibits the activation of NF-κB as it suppresses the degradation of IκBα and the translocation of p65 from the cytosol to the nucleus (Pradeep and Kuttan, 2004; Chung, 2019; Yang et al., 2019). Additionally, recent docking studies revealed that piperine exhibits significant binding affinity toward the spike glycoprotein of SARS-CoV-2 and the ACE2 receptor. Hence, piperine may prove to be a useful therapeutic agent not only for restricting attachment of virus to the host cells but also for targeting the cytokine storm by suppressing the activity of NF-κB and MAPK pathways and subsequent pro-inflammatory cytokines release (Maurya et al., 2020).

### Quercetin

Quercetin (2-(3,4-dihydroxyphenyl)-3,5,7-trihydroxychromen-4-one) is a polyphenolic flavonoid found in abundance in many plants including broccoli, red onions, eggplant, potatoes and green leafy vegetables including celery, lettuce; fruits including apples, citrus fruits, red grapes, tomatoes; berries including cranberries and raspberries. The extract of quercetin has been used for prevention and treatment of rheumatic disease, cardiovascular disease, hypercholesterolemia, infections and cancers (Li et al., 2016; National Center for Biotechnology Information, 2020l). Quercetin was found to effect the production of cytokines; it was found to decrease the production of pro-inflammatory cytokines TNFα, IL-6, G-CSF, GM-CSF and VEGF and chemokines IP10 and MCP1 in mouse macrophages induced with polyinosinic-polycytidylic acid, and bring about an increase in the level of anti-inflammatory cytokine IL 27 in influenza A-treated MDCK cells (Kim and Park, 2016; Mehrbod et al., 2018). It was also found to stimulate T-helper cells to produce IFNγ and downregulate Th2-derived IL-4 in cultured blood peripheral mononuclear cells (Biancatelli et al., 2020). Carrageenin-induced production of IL-1β was significantly reduced with quercetin treatment (Valério et al., 2009). Quercetin was responsible for a marked reduction in the production of TNFα, in a dose-dependent manner in a study in normal PBMC. This inhibitory effect of quercetin on the pro-inflammatory cytokine TNFα is mediated via the modulation of NF-κB1 and IκB (Nair et al., 2006). Quercetin treatment was found to bring about a reduction in the LPS-induced increasing levels of inflammatory factors IL-1β, IL-6 and TNFα in RAW 264.7 cells. The ability of quercetin to suppress the production of pro-inflammatory cytokines is mediated by its suppression of the activation of ERK and p38 MAP kinase and NF-κB/IκB signal transduction pathways (Tang et al., 2019). In a study on LPS-stimulated RAW 264.7 cells, quercetin decreased the activation of phosphorylated ERK kinase and p38 MAP kinase. No significant effect on JNK MAP kinase was observed. Additionally, quercetin also inhibited the activation of NF-κB/IκB complex and the degradation of IκB (Cho et al., 2003). Quercetin brought about a dose-dependent reduction in the levels of IL-6, IL-8, MCP1 and ICAM1 in IL-1β-stimulated human retinal pigment epithelial (ARPE-19) cells. Quercetin was found to inhibit the signaling pathways related to the inflammatory process; including inhibition of the phosphorylation of mitogen-activated protein kinases (MAPKs), inhibiting the nuclear factor κ-B kinase (IKK)α/β, c-Jun, cAMP response element-binding protein (CREB), activating transcription factor 2 (ATF2) and nuclear factor (NF)-κB p65, and blocking the translocation of NF-κB p65 into the nucleus (Cheng et al., 2019). Another study on patients with coronary artery disease showed that treatment with quercetin brought about a reduction in the levels of IL-1β, TNFα and the levels of IL-10 tended to decrease. This was attributed to a decrease in the transcriptional activity of NF-κB (Chekalina et al., 2018). Oral supplementation with quercetin treatment is relatively safe, causing no significant adverse effects; headache and temporary peripheral paresthesia were the only effects observed in two members out of thirty in a study. The use of quercetin as an adjuvant to concurrently fortify the immune response by promoting IFNs production and modulating the levels of pro- and anti-inflammatory cytokines may complement currently used interventions in treating COVID-19 (Biancatelli et al., 2020).

### Resveratrol

A polyphenolic phytoalexin, resveratrol (5-[(E)-2-(4-hydroxyphenyl)ethenyl]benzene-1,3-diol) is found in more than 70 species of plants, such as grapes, cranberry, blueberry, mulberry, peanuts, jackfruit, soy and wine. Resveratrol is known to posses a number of beneficial health effects; it is has antioxidant, anticancer, anti-inflammatory, antiviral, anti-aging and life-prolonging effects (Bansal et al., 2018; National Center for Biotechnology Information, 2020m). Resveratrol is an efficient immunomodulator; it interferes with immune cell regulation, pro-inflammatory cytokine synthesis and gene expression, thus regulating immunity (Malaguarnera, 2019). In a study carried out, resveratrol caused an irreversible inhibition of IFNγ and IL-2 by splenic lymphocytes and the production of TNFα and IL-12 by peritoneal macrophages. The activation of NF-κB was blocked without affecting the basal NF-κB activity (Gao et al., 2001). In another study inflammatory cytokine and chemokine release (GM-CSF and CXCL8) by stimulated alveolar macrophages from COPD patients was blocked by resveratrol (Culpitt et al., 2003). Resveratrol blocks the increased secretion of IL-6 and TNFα in EV71-infected RD cells (Zhang et al., 2015). In yet another study, treatment with resveratrol induced a dose-dependent inhibition of IL-1α, IL-6 and TNFα *in vitro* and downregulated the production of IL-17 in HTLV-1 infected cells (Fuggetta et al., 2016). Resveratrol treatment was found to be associated with downregulation of NF-κB in the inflammatory cells of the lungs (Rieder et al., 2012). LPS-induced phosphorylation and degradation of IκBα is blocked in macrophages subject to resveratrol treatment decreasing the NF-κB DNA binding activity. The inhibitory effect of resveratrol on the NF-κB pathway correlates with its ability to bring about a reduction in IKK activity (Yamamoto and Gaynor, 2001). In a study evaluating the efficacy of resveratrol dry suspension in pigs, it was found that resveratrol upregulated the release of IFNγ and downregulated the release of TNFα; thus regulating the humoral immune responses (Fu et al., 2018). A dose-dependent reduction in the levels of pro-inflammatory cytokines IL-6, IL-8 and MCP1 was observed with resveratrol treatment in adipocytes under inflammatory conditions (Zagotta et al., 2015). The inhibitory effect of resveratrol on TNFα, IL-6 and MIP2 levels was established in a study on rabbit model of acute pharyngitis. Resveratrol reduced the protein expression of IL-1β and IL-18. Furthermore, treatment with resveratrol resulted in suppression of TLR4 and myeloid differentiation primary response protein 88 protein expression, reduced p-NF-κB and increased *p*-IκB protein expression (Zhou et al., 2018). A decreased production of cytokines IL-1β, IL-6, IL-8, IL-12 and TNFα was observed on treatment with resveratrol in a dose- and time-dependent (Rizzo et al., 2012). Resveratrol is considered safe when taken at supplemental doses. Resveratrol can be an adjunctive agent to consider for SARS-CoV-2 infection, to mitigate the cytokine storm and consequently reduce inflammation (Marinella et al., 2020).

A few more plant-derived immunosuppressants that can be potentially used as adjuvants in COVID-19 therapy are described in Table 1.

**TABLE 1:** Plant-derived Immunosuppressants and their Effect on Pro-inflammatory Cytokines.

S. No	Natural Immunomodulatory Agent	Plant Source	Immuno-Action	Effect on Cytokines	Probable Mechanism of Action	Study	References
1	Citral	Citrus fruits, lemongrass	Immunosuppressant	Inhibits IL-1β and IL-6 release, IL-10 production	Inhibition of NF-κB	Macrophages challenged with LPS	Bachiega et al. (2011)
				Reduced IL-1β, IL-4, INFγ, TNFα production; inhibits NLRP3 inflammasome activation	Inhibits ATP-induced caspase-1 activity; inhibits NF-κB, p65 activation	LPS-induced mouse ASLN model	Ka et al. (2015)
2	Ginsenoside	Panax ginseng C.A.Mey., P. notoginseng (Burkill)F.H.Chen., P. quinquefolius L., *P. japonicus* var. major (burkill) C.Y.Wu. and feng (and other members of panax genus)	Immunosuppressant	Reduced TNFα, IL-6, IL-1β, IL-12p40, CXCL2 mRNA expression; augmented IL-10 expression	Inhibits NF-κB and MAPK pathways	LPS-activated BMDMs	Paik et al. (2019)
				Downregulates IL-6, TNFα and IL-10 expression	Inhibits NF-κB activation	II/R induced lung injury *in vivo*	Jiang et al. (2014)
3	Kaempferol	Delphinium, witch-hazel, grapefruit, and other plant sources	Immunosuppressant	Reduced overproduction of TNF α, IL-1β, IL-6, ICAM1, VCAM1	Negative regulation in TLR4, NF-κB and STAT signaling	LPS-induced rat intestinal microvascular endothelial cells (RIMVECs)	Bian et al. (2019)
				Inhibited release of IL-6, IL-8 and TNFα	Inhibited activation of PKC *θ*	C57BL/6 anti-BDF1 MLC	Kempuraj et al. (2005)
4	Withaferin A	Withania somnifera (L.) Dunal and other members of solanaceae family	Immunosuppressant	Inhibits IL-1α, IL-1β, IL-2, IL-3, IL-5, IL-10, IL-12p70, IL-13, IL-18, IP10, GM-CSF, CCL2/MCP-1, CCL17/TA RC, SDF1α/CXCL12 and CCL20/MIP3α	Targets NF-κB and the inflammasome complex (blocks the nuclear translocation of NF-kB; inhibits caspase-1 activation)	ATP-stimulated monocyte-derived THP-1 cells	Dubey et al. (2018)
				Dose-dependent reduction in IL-6, IL-8, IL-1α, MIP1α, MIP1β, G-CSF and IP10	NF-κb inhibition	Mouse and human islet cells; *in vitro*	SoRelle et al. (2016)

## Conclusion

The COVID-19 pandemic is affecting millions of people across the globe. In some patients infected with SARS-CoV-2, the immunological reaction involves mobilization and sustained production of several pro-inflammatory cytokines and chemokines, giving rise to a cytokine storm. This cytokine storm appears to be one of the common causes of mortality in this disease. This necessitates a suitable therapeutic strategy that can efficiently attenuate this excessive host immune response. Different synthetic immunomodulatory agents such as corticosteroids, interleukin receptor antagonists, JAK/STAT inhibitors and monoclonal antibodies like tocilizumab, sarilumab, infliximab and adalimumab that target specific cytokines are being evaluated for treatment of COVID-19 patients. Unfortunately, most of these immunomodulators are known to cause adverse effects, thus limiting their use. Plant-derived immunosuppressants present an invaluable alternative to manage the cytokine storm syndrome observed in COVID-19. They have a good safety profile and specifically target the production and release of certain cytokines by suppressing definite reactions in the signaling cascades. Additionally, some of these agents also exhibit antiviral properties against coronaviruses and some others can potentially inhibit the main protease of SARS-CoV-2. Clinical trails to investigate the dosage, administration timing, bioefficacy, bioavailability, mechanism of action and safety with respect to COVID-19 is the need of the hour to capitalize on this promising therapeutic approach. A combination treatment including plant-derived immunosuppressants along with antiviral agents and other drugs will be effective in tackling SARS-CoV-2 infection, rather than the use of a single drug alone. Such a synergistic therapeutic approach with the inclusion of natural immunosuppressants to ameliorate the cytokine storm holds great treatment potential.

## Glossary

ACE: Angiotensin-converting enzyme
AMPK: 5’AMP-activated protein kinase
ARDS: Acute respiratory distress syndrome
ASLN: Accelerated and severe lupus nephritis
BMDM: Bone-marrow derived macrophage
COPD: Chronic obstructive pulmonary disease
COVID-19: Coronavirus disease 2019
COX: Cyclooxygenase
CRP: C-reactive protein
DSS: Dextran sulfate sodium
EAC: Ehrlich ascites carcinoma
ELISA: Enzyme-linked immunosorbent assay
ESR: Erythrocyte sedimentation rate
FMF: Familial Mediterranean fever
FLS: Fibroblast-like synoviocytes
G-CSF: Granulocyte colony-stimulating factor
GM-CSF: Granulocyte-macrophage colony-stimulating factor
HGF: Hepatocyte growth factor
HIV: Human immunodeficiency virus
HMC: Human mast cell line
ICAM: Intercellular adhesion molecule
IFN: Interferon
II/R: Intestinal ischemia and reperfusion
IkBα: I-kappa B-alpha
IKKβ: Inhibitor of nuclear factor kappa-B kinase subunit beta
IL: Interleukin
iNOS: Inducible nitric oxide synthase
IP: Interferon gamma-induced protein
IRF: Interferon regulatory factor
IVIG: Intravenous immunoglobulin
JAK: Janus kinase
LPS: Lipopolysaccharide
MAPK: Mitogen activated protein kinase
MASP: Mannose-binding lectin serine protease
MCP: Monocyte chemoattractant protein
MCSF: Macrophage colony-stimulating factor
MIG: Monokine induced by gamma; CXCL9
MIP: Monocyte chemoattractant protein
MOF: Multiple organ failure
MSC: Mesenchymal stem cell
MSU: Monosodium urate
NADPH: Nicotinamide adenine dinucleotide phosphate
NF-κB: Nuclear factor kappa-light-chain-enhancer of activated B cells
NK: Natural killer
NLR: NOD-like receptor
NOX2: NADPH oxidase 2
NOD: Nuclear-binding and oligomerization domain
OVA: Ovalbumin
PAMP: Pathogen-associated molecular pattern
PBMC: Peripheral blood mononuclear cells
PCT: Procalcitonin time
PDGF: Platelet-derived growth factor
pERK: Phosphorylated extracellular signal-regulated kinase
PF: Pulmonary fibrosis
PGE2: Prostaglandin E2
pJNK: Phosphorylated c-Jun N-terminal kinase
PKC: protein kinase C
PMA: Phorbol 12-myristate 13-acetate
pMACO: Permanent middle cerebral artery occlusion
PT: Prothrombin time
RA: Rheumatoid arthritis
RBD: Receptor binding domain
RD: Rhabdosarcoma
rhACE: recombinant hACE
RIG: Retinoic acid-inducible gene I
RLR: Rig-I-like receptor
RNA: Ribonucleic acid
RT-PCR: Reverse transcription polymerase chain reaction
SARS-CoV-2: Severe acute respiratory syndrome coronavirus 2
STAT: Signal transducer and activator of transcription
TLR: Toll-like receptor
TNBS: 2,4,6-trinitrobenzene sulfonic acid
TNF: Tumor necrosis factor
VEGF: Vascular endothelial growth factor
